# An approximation of herd effect due to vaccinating children against seasonal influenza – a potential solution to the incorporation of indirect effects into static models

**DOI:** 10.1186/1471-2334-13-25

**Published:** 2013-01-22

**Authors:** Ilse Van Vlaenderen, Laure-Anne Van Bellinghen, Genevieve Meier, Barbara Poulsen Nautrup

**Affiliations:** 1 CHESS, Kerkstraat 27, 1742, Ternat, Belgium; 2 Health Economics, GlaxoSmithKline Vaccines, 2301 Renaissance Boulevard, RN 0220, King of Prussia, PA, 19406, USA; 3 EAH-Consulting, Heimbacher Str. 19, 52428, Juelich, Germany

**Keywords:** Paediatric, Vaccination, Influenza, Herd protection, Herd effect, Herd immunity, Modelling, Economic evaluation

## Abstract

**Background:**

Indirect herd effect from vaccination of children offers potential for improving the effectiveness of influenza prevention in the remaining unvaccinated population. Static models used in cost-effectiveness analyses cannot dynamically capture herd effects. The objective of this study was to develop a methodology to allow herd effect associated with vaccinating children against seasonal influenza to be incorporated into static models evaluating the cost-effectiveness of influenza vaccination.

**Methods:**

Two previously published linear equations for approximation of herd effects in general were compared with the results of a structured literature review undertaken using PubMed searches to identify data on herd effects specific to influenza vaccination. A linear function was fitted to point estimates from the literature using the sum of squared residuals.

**Results:**

The literature review identified 21 publications on 20 studies for inclusion. Six studies provided data on a mathematical relationship between effective vaccine coverage in subgroups and reduction of influenza infection in a larger unvaccinated population. These supported a linear relationship when effective vaccine coverage in a subgroup population was between 20% and 80%. Three studies evaluating herd effect at a community level, specifically induced by vaccinating children, provided point estimates for fitting linear equations. The fitted linear equation for herd protection in the target population for vaccination (children) was slightly less conservative than a previously published equation for herd effects in general. The fitted linear equation for herd protection in the non-target population was considerably less conservative than the previously published equation.

**Conclusions:**

This method of approximating herd effect requires simple adjustments to the annual baseline risk of influenza in static models: (1) for the age group targeted by the childhood vaccination strategy (i.e. children); and (2) for other age groups not targeted (e.g. adults and/or elderly). Two approximations provide a linear relationship between effective coverage and reduction in the risk of infection. The first is a conservative approximation, recommended as a base-case for cost-effectiveness evaluations. The second, fitted to data extracted from a structured literature review, provides a less conservative estimate of herd effect, recommended for sensitivity analyses.

## Background

Influenza is an acute viral infection. While self-limiting in most people, it can result in serious illness or death in certain high-risk groups, such as elderly people (aged 65 years or more), young children (aged 2 years or less), or people with chronic medical conditions. The clinical and economic burden of influenza is substantial. In the United Kingdom (UK), influenza has been estimated to account for 779,000 to 1,164,000 general practitioner (GP) consultations, 19,000 to 31,200 hospital admissions and 18,500 to 24,800 deaths annually [1]. A study analysing US Medicare data over six influenza seasons estimated the average cost of influenza-associated hospitalisations in elderly patients at $372 million per year [2].

Vaccination is the most effective way to prevent influenza infection [3], and annual vaccination of high-risk groups is recommended by the World Health Organization (WHO) [3] and implemented in many countries [4,5]. However, high-risk groups may be difficult to reach for influenza vaccination [6], and the immune response to influenza vaccination has been reported to be lower in elderly people than in younger adults [7]. Thus, vaccination only of high-risk groups may not maximise overall health benefits. Vaccination of other population groups may offer a way to improve protection in high-risk groups via herd protection effects, whereby vaccination of one part of the population confers partial indirect protection against infection for the unvaccinated remainder by reducing the circulation of the virus within the population [8]. According to the current concept children are the main disseminators of influenza both in the household and the entire community during local outbreaks [9]. Evidence indicates that vaccination of this specific population against influenza has the potential to provide indirect benefits to the entire community, including high-risk and elderly populations [8,10,11]. For example, a study in Canada found that vaccination of children and adolescents up to age 15 years against influenza achieved a protection of 61% against influenza infection in unvaccinated individuals [11].

Herd effect may thus be an important component of the public health effects of influenza vaccination. Economic evaluations of influenza vaccination that take account of herd effect will be needed by healthcare decision-makers appraising influenza vaccination programmes, in order to capture fully the direct and indirect benefit of childhood vaccination. Static models are most often used to evaluate the cost-effectiveness of mass vaccination against seasonal influenza, whereas dynamic models are most often used to evaluate the impact of vaccination on transmission and disease incidence. However, static cohort models cannot dynamically capture the effect of vaccination on transmission and therefore fail to account for herd effect [12,13]. As such, static models generally underestimate the total reduction in the number of incident cases likely to result from vaccination.

If herd effect is included in static models, most use a fixed input parameter derived from empirical data, such as the reduced incidence in susceptible individuals at a specific, pre-defined vaccination coverage [14]. However, this approach does not allow the simulation of varying levels of vaccination coverage in different target population groups or for the impact of varying vaccine efficacy, which is – amongst others – dependent on age, degree of strain matching and type of vaccine [15]. If the indirect benefits of vaccinating varying proportions of children with varying efficacious vaccines are to be incorporated in a static model, this will need a non-dynamic approximation of the relationship between effective coverage in these children and the respective reduction in the risk of infection in the rest of the community.

The objective of the current study was to develop an approximation to capture the herd effect induced by annual vaccination of children against influenza at varying coverage levels. This approximation can be incorporated into cohort models to permit the consideration of indirect benefits for the community achieved by annual vaccination of children, without the need to rely on dynamic modelling processes.

## Methods

### Definitions

Throughout this manuscript, the following definitions apply:

Effective coverage = vaccination coverage × vaccine efficacy

Effective coverage in children = vaccination coverage in children × vaccine efficacy in children

Change in effective coverage in the entire population (induced by effective coverage in children) = effective coverage in children × proportion of children in the total population

### Linear approximation of herd effect

Bauch et al. (2009) [12] describe a pseudo-dynamic approximation to allow incorporation of herd effect in a focal cohort (vaccinated in year X), induced by vaccinating subsequent cohorts not included in the focal cohort model (vaccination in years X + 1, X + 2, X + 3, etc.). Equations 2 and 3 in this publication estimate an adjustment factor ω by which the incidence in susceptible individuals should be multiplied to capture partial herd effect benefits. Both equation functions are linear. Assuming ω is a good approximation of the relative risk (RR) of infection induced by herd effect, the linear relationship between RR and effective coverage estimated from Equations 2 and 3 is presented in a figure in the publication by Bauch et al. (2009) [12] (the second figure in Bauch et al. (2009) [12]).

### Derived from Equation 2 (known R_0_)

$$\text{RR}\approx \frac{R_{0}\left(1-\text{effective}\;\text{coverage}\right)-1}{R_{0}-1}$$

R_0_ = basic reproduction number (average number of secondary infectious persons resulting from the introduction of an infectious person into a totally susceptible population).

The RR of infection can be described as decreasing linearly with increasing effective coverage. The slope of the line (or the value of effective coverage at which a RR of zero is achieved, i.e. the elimination threshold) is dependent on the value of R_0_: the lower the R_0_, the steeper the decrease in RR, i.e. the higher the impact of herd effect (the second figure in Bauch et al. (2009) [12]). Detailed information on the relationship between R_0_ and the magnitude of herd effect can be found in Bauch et al. (2009) [12].

### Derived from Equation 3 (unknown R_0_)

RR≈1−effectivecoverage

This equation is only dependent on effective coverage, and is the most conservative approach for estimating the relationship between effective coverage and the RR of infection, since it does not account for any incremental herd immunity induced by R_0_ approaching 1 (the second figure in Bauch et al. (2009) [12]).

Although Bauch et al. (2009) [12] suggest a linear approximation of herd effect, their settings and assumptions deviate substantially from those generally accepted for seasonal influenza. Further confirmation was required on whether a linear approximation could also be considered valid for annual vaccination against seasonal influenza and therefore this was the rationale for conducting a literature review to identify published evidence to test this hypothesis.

### Structured literature review with a focus on seasonal influenza

A structured literature review was performed with a specific focus on herd effect induced by vaccination against seasonal influenza. The objectives of this review were: to validate whether a linear relationship between effective coverage in a subpopulation and RR of symptomatic influenza infection in the non-vaccinated population forms a valid approximation for herd effect; and to identify point estimates of this relationship, expressed as RR as a function of effective coverage in children. Methods of analysis, i.e. keywords, limitations, inclusion criteria, as well as the data extraction sheet, were defined *a priori*.

### Database search

Free-text PubMed searches were conducted using the following search terms, limited to English-language publications in humans with abstracts available:

1. influenza

2. herd immunity OR herd protection OR herd effect

3. population protection OR community protection

4. community vaccination OR community disease transmission

5. 1 AND (2 OR 3 OR 4)

No time limits were applied. The last search was run on 3 August 2011.

### Other searches

Relevant references cited in articles identified through the database search, as well as literature identified from other sources, were included. Literature identified through other sources was clearly stated as such, as these may be subject to search bias.

### Eligibility criteria

Articles were included if they met the following pre-defined criteria:

1. Clinical study or observational study or review or modelling or health economic study;

2. Inclusion of a subpopulation for mass vaccination;

3. Reporting of one of the following outcomes (either directly reported, or reported outcomes allowing a recalculation to obtain these data):

a. A relationship (mathematical function) between varying degrees of vaccine coverage and efficacy in subgroup populations (not restricted to children) and the reduction of influenza transmission (i.e. reduction in probability of infection) in a larger unvaccinated population;

b. Point estimates of the reduction of influenza infection in the unvaccinated population after vaccination of children, which allow for a fitting of the mathematical function to published data (as defined under (a)).

Titles and abstracts were scanned, and the full text of publications meeting the eligibility criteria or requiring further evaluation was reviewed. Publications meeting the eligibility criteria after evaluation of the full text were included in the full data extraction process.

### Data extraction

The data extraction sheet was pre-defined and only minor changes, mainly to improve clarity, were applied after the start of review. Data extraction was conducted by one reviewer and reassessed by an independent reviewer (included studies only). Any discrepancies, which were only minor and non-substantial, were resolved by discussion between the two reviewers.

### Outcomes considered and additional analyses

The main outcomes and additional analyses from the publications included in the literature review were as follows:

• Vaccination coverage and direct effectiveness of vaccination in subgroup population;

Additional analysis (if not reported): calculation of effective coverage in subpopulation, based on vaccination coverage in subpopulation and effectiveness expressed as a reduction in the probability of infection in vaccinated individuals;

• Indirect effectiveness in unvaccinated individuals after vaccination of subpopulations;

Additional analysis (if not reported): calculation of the reduction in probability of infection in the unvaccinated population, based on the probability of infection in the absence (or baseline level) of effective coverage in subpopulations, and the probability of infection in the presence of increased effective coverage in subpopulations;

• Relationship (mathematical function and point estimates) between different levels of effective coverage in subpopulation and indirect effectiveness in unvaccinated individuals after vaccination of subpopulation;

Additional analysis (if not reported): calculated relationship (mathematical function) between different levels of effective coverage in subpopulation and changes in RR in unvaccinated population.

### Function fitting process

The linear function calculated from Equation 3 in Bauch et al. (2009) [12] did not contain a fitting parameter and hence was not fitted to the point estimates. This function accounts only for the reduction in the number of susceptible individuals due to vaccination, and can therefore be applied to estimate on a yearly basis the RR for seasonal influenza infection.

In a second approach, a linear function was fitted to the point estimates identified through the structured literature review as best predictors of the functional relationship between effective coverage in children and RR of infection in the unvaccinated remainder of the population. Theoretically, the linear function calculated from Equation 2 in Bauch et al. (2009) [12] could have been used for this purpose; R_0_ would then be the fitting parameter. However, Equation 2 in Bauch et al. (2009) [12] was developed for a particular situation, in which – amongst others – natural and vaccine-derived immunity are lifelong. Since this is not the case for seasonal influenza, any value attributed to R_0_ as a result of the fitting process would be of no epidemiological meaning.

Therefore, a simple linear function of the form *y* = *a* + *bx* was fitted to the point estimates identified through the structured literature review as best predictors, by minimizing the sum of squared residuals (SSR) using the methodology described by Kemmer and Keller (2010) [16]. In this function, y = RR, x = effective coverage, a = 1 (ensuring RR = 1 at zero per cent effective coverage), and b = fitting parameter. The slope and intercept of the resulting linear function obtained by this fitting process are identical to those that would have been obtained by applying Equation 2 in Bauch et al. (2009) [12], but there is no epidemiological meaning attributable to the fitting parameter b.

## Results

### Structured literature review

**Figure 1:**
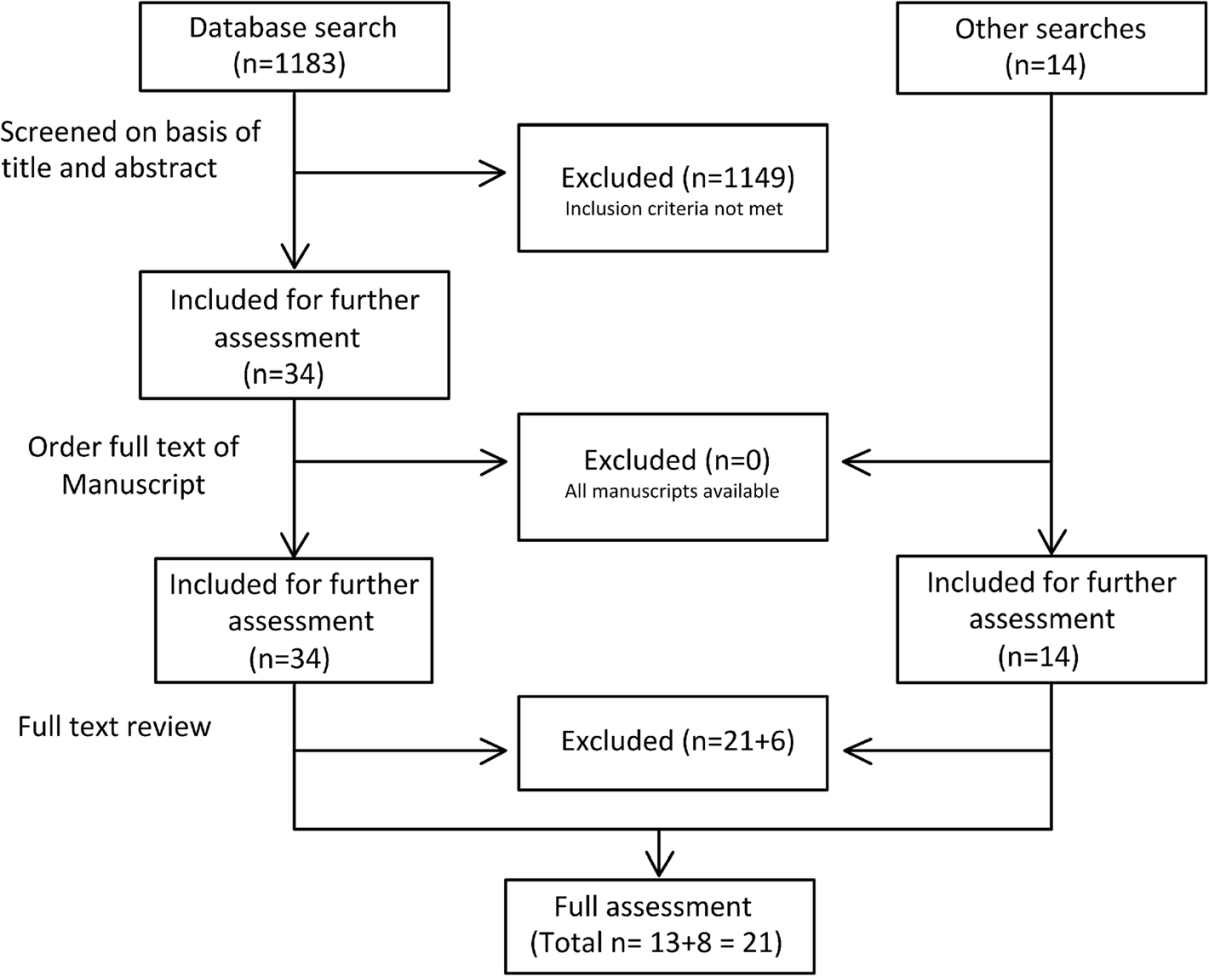
Flow diagram for the literature review.

Figure 1 summarises the study selection process. After full text review, a total of 27 studies (21 identified through the database search and 6 from other sources) were excluded. The reasons for exclusion were: reviews with descriptive analyses only (n = 7); Cochrane review not meeting the inclusion criteria for outcomes reported (n = 1); meeting report summarising results reported elsewhere (n = 1); no inclusion of subgroup population for mass vaccination (n = 1); inclusion criteria for outcomes reported not fulfilled (n = 17).

### Studies included

A total of 21 publications were included, two of which [17,18] reported the same clinical trial, resulting in a total of 21 publications on 20 studies. Eight studies reported data useful for the estimation of a mathematical function [13,19-25], 8 studies reported point estimate data on herd effect at a community level [11,23-29] and 8 studies in 9 publications reported point estimate data on herd effect in subpopulations [17,18,21,30-35]. These studies are summarised in Table 1.

**Table 1:** Overview of studies included

**Study**	**Source**	**Type of study**	Outcomes reported as relevant for model population*
Clover et al. (1991) [30]	Other searches	Trial	Point estimates
Elveback et al. (1976) [23]	Other searches	Model	Mathematical function deducible
			(Point estimates)
Esposito et al. (2003) [31]	Other searches	Trial	Point estimates
Ghendon et al. (2006) [32]	Database	Trial	Point estimates
Glezen et al. (2010) [26]	Database	Trial	(Point estimates)
Gruber et al. (1990) [33]	Other searches	Trial	Point estimates
Halloran et al. (2002) [24]	Database	Model	Mathematical function deducible
			Point estimates
Hurwitz et al. (2000) [34]	Other searches	Trial	Point estimates
Lemaitre et al. (2009) [20]	Database	Trial	(Mathematical function)
Loeb et al. (2010) [11]	Database	Trial	Point estimates
Milne et al. (2010) [19]	Database	Model	(Mathematical function)
Monto et al. (1969) [17]	Database	Trial (both articles reporting the same trial)	Point estimates
Monto et al. (1970) [18]	Other searches		
Piedra et al. (2007) [28]	Database	Trial	(Point estimates)
Piedra et al. (2005) [27]	Database	Trial	(Point estimates)
Pradas-Velasco et al. (2008) [13]	Database	Model	Additional information on the mathematical function
Principi et al. (2003) [35]	Other searches	Trial	Point estimates
Rudenko et al. (1993) [21]	Other searches	Trial	Mathematical function
			Point estimates
Van den Dool et al. (2008) [22]	Database	Model	Mathematical function
Vynnycky et al. (2008) [29]	Database	Model	Point estimates
Weycker et al. (2005) [25]	Database	Model	Mathematical function deducible
			(Point estimates)			

* Outcomes assessed as not useful for the current study are given in parentheses.

### Studies reporting data useful for the estimation of a mathematical function

The first aim of the literature review was to identify studies that allowed us to test whether a linear relationship between varying degrees of effective coverage in subgroup populations and the reduction of risk of influenza infection in a larger unvaccinated population was a plausible assumption for annual seasonal influenza vaccination. Eight studies identified in the review reported a mathematical function, allowed the recalculation of data and creation of a graph, or provided other data relevant to this aim. Of these, two were not further considered because the function could not be solely attributed to indirect effects [19] or because only a graphical depiction of the correlation between staff vaccination coverage and all-cause mortality rates in residents of nursing homes was reported [20] (Table 1).

**Figure 2:**
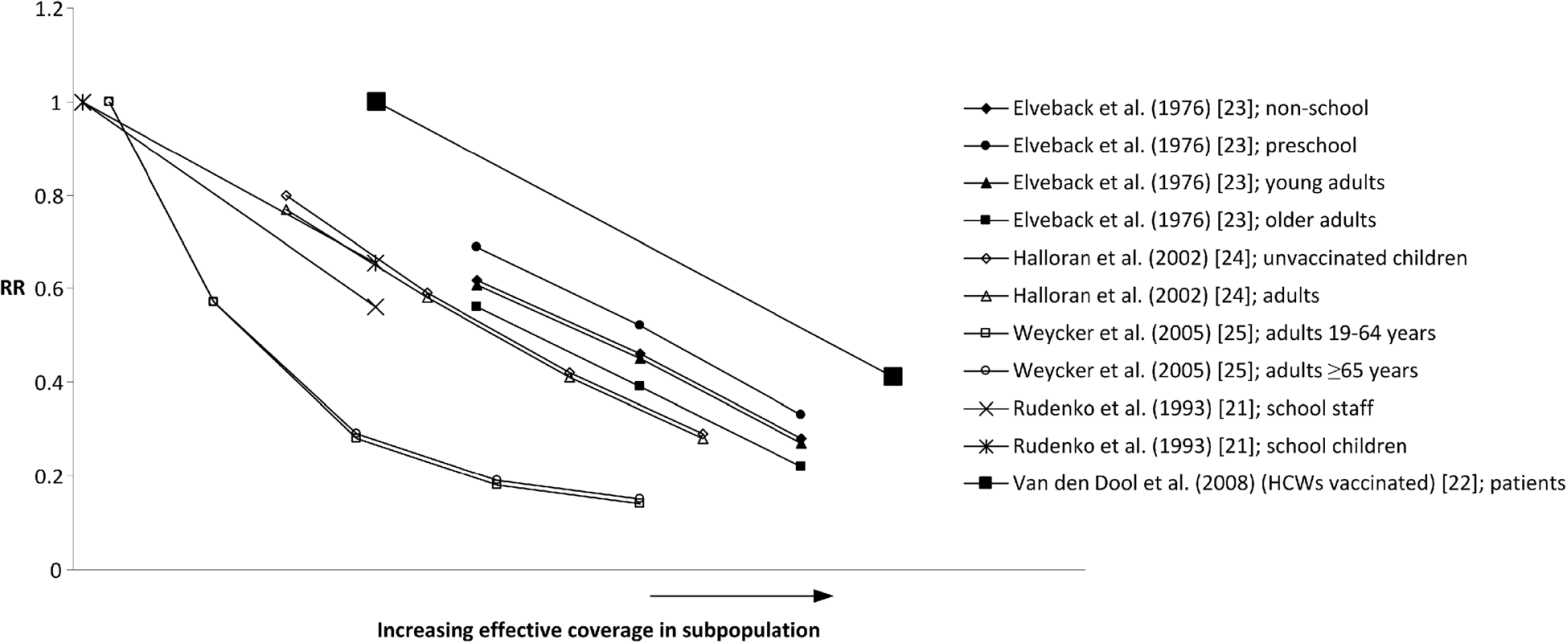
**Graphical relationships between vaccine coverage and herd effect in published studies.** Relationship between effective vaccine coverage in subpopulation and relative risk of influenza infection in the population analysed for herd effect. Based on data from five studies [21-25]. Absolute values of the different studies reported in this figure cannot be compared. HCW, healthcare workers.

Of the remaining six studies, two provided a graphical illustration [21,22], three reported data that allowed the estimation of a graphical illustration [23-25] and one provided other relevant data [13]. The studies included are described in more detail in Additional file 1. The graphs derived from these studies are shown in Figure 2. Two dynamic population models [23,24] resulted in linear relationships over the range of vaccine coverage reported in the studies. Another dynamic population model [25] resulted in an exponential function (with exponent <1) for a range of effective coverage between 3.5% and 56%. A cluster randomized clinical trial calculated slopes between the percentage of children vaccinated and staff illness, as well as illness rate of unvaccinated students in the same school [21]. One study reported and graphically depicted a strong linear relationship between patients’ attack rates and varying levels of effective coverage in health care workers [22] (Figure 2). It should be noted that the absolute values of the different studies reported in Figure 2 cannot be compared, because the studies included different subpopulations (children, healthcare workers) and in one study [21] the original study reported a slope for increasing vaccine coverage, which we have applied to effective coverage in Figure 2. Thus, the absolute values of the point estimates reported for this study in Figure 2 are not accurate, but the linear relationship is still valid. One study, comparing a static and a dynamic model, revealed that with low levels of effective coverage a high percentage of the total vaccination effect is due to herd effect [13] (see Additional file 1 for more details).

Overall, the studies reporting data useful for estimating mathematical functions suggested that within an effective coverage range (vaccine efficacy combined with coverage) of 20% to 80% of the subgroup targeted for vaccination, there was evidence for a linear relationship between effective coverage and RR. For very low effective coverage levels (<20%), literature did not reveal a mathematical function for the relationship between effective coverage and relative risk. However, findings indicate that herd effect is relevant even with very low levels of coverage and can be even greater than direct effect [13]. No information was identified from the literature on changes to the RR in unvaccinated persons with high levels of effective coverage (>81.1%) in vaccinated subpopulations.

### Studies reporting point estimates

The second aim of the literature review was to identify point estimates for the reduction of influenza infection in the unvaccinated population after vaccination of children, which can be used to populate the linear mathematical function defined from literature. A total of 16 articles on 15 studies reported point estimates or allowed the recalculation of point estimates on the reduction of influenza incidence in the unvaccinated population after vaccination of children. The herd effect was evaluated either at the community level (8 studies [11,23-29]) or within a subpopulation (8 studies reported in 9 articles [17,18,21,30-35]). However, from one of the subpopulation studies, point estimates for a herd effect in school contacts could only be approximated [21].

Of the studies evaluating herd effect at a community level, five were considered unsuitable for estimation of point estimates. Three studies [26-28] were all part of the Central Texas Field Trial and used the outcome parameter ‘medically attended acute respiratory illness’ (MAARI), which has considerable limitations, and also had other methodological issues (see Additional file 1 for more details). One study only allowed the recalculation of RR to a reference (RR = 1.00) that corresponded to 3.5% effective coverage rather than zero coverage [25], and the fifth study was validated for pandemics rather than epidemics [23].

Table 2 shows the results for point estimates, recalculated to effective coverage and RR of infection, from the three studies considered suitable for the identification of point estimates on the reduction of influenza incidence in the unvaccinated population after vaccination of children [11,24,29]. All three studies considered influenza (laboratory-confirmed in the clinical study [11], and confirmed influenza as considered in the modelling studies [24,29]), rather than influenza-like illness. Descriptions of the three studies and the age groups evaluated are included in Additional file 1. The table shows RR for each study and population at varying levels of effective coverage in children (row A).

**Table 2:** Point estimates for relationship between risk of infection in unvaccinated population and vaccine coverage

**A. Effective coverage in children**	**0.0%**	**21.00%**	**35.00%**	**45.65%**	**49.00%**	**60.00%**	**62.30%**
**Proportion of children in the total population, for estimating B.**		**25.78% **[24]	**25.78% **[24]	**35.70% **[11]	**25.78% **[24]	**21.08% **[29]	**25.78% **[24]
**B. Change in effective coverage in entire population (induced by varying levels of effective coverage in children)**	**0.0%**	**5.41%**	**9.02%**	**16.30%**	**12.63%**	**12.65%**	**16.06%**
**Study and population analysed**							
Vynnycky et al. (2008) [29]	1.00					0.44	
Influenza A, 15–44 years, minimum							
Vynnycky et al. (2008) [29]	1.00					0.05	
Influenza A, 15–44 years, maximum							
Loeb et al. (2010) [11]	1.00			0.39			
Entire (unvaccinated) population							
Halloran et al. (2002) [24]	1.00	0.80	0.59		0.42		0.29
Unvaccinated children							
Halloran et al. (2002) [24]	1.00	0.77	0.58		0.41		0.28
Adults							
**RR estimates from fitted general linear equation**							
A. In unvaccinated remainder of children *	1.00	0.75	0.58	0.45	0.41	0.28	0.25
B. In other age groups **	1.00	0.75	0.58	0.24	0.41	0.41	0.25

Point estimates for relationship between relative risk of infection in unvaccinated population as a function of (A) effective coverage in children, and (B) change in effective vaccine coverage in entire population induced by varying levels of effective coverage in children, and the corresponding RR estimates from the fitted general linear equations.

*RR_*unvaccinated children*_ = 1–1.2031*effective coverage in children.

**RR_*other age groups*_ = 1–4.6656*(*effective coverage in children*)* *P*_*children*_.

**Figure 3:**
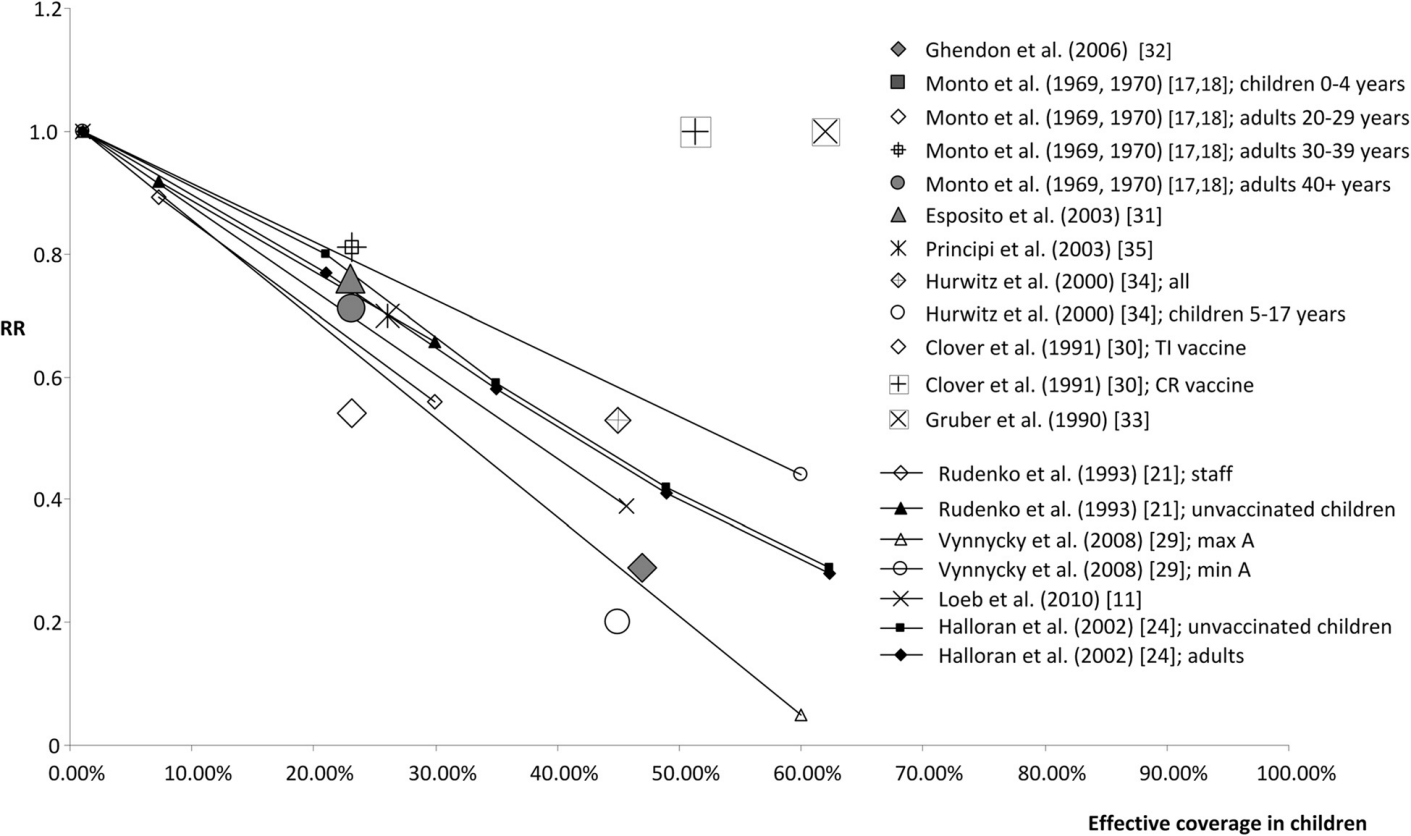
**Point estimates from studies evaluating herd effect in a subpopulation in published studies.** Single data points show point estimates of relative risk (RR) of influenza infection in subpopulation analysed for herd effect plotted against effective vaccine coverage in children. Point estimates from studies evaluating herd effects at a community level are shown as lines (derived by connecting lines through the point where RR = 1.0 and effective coverage = 0%) for comparison.

 Eight studies provided information on herd effects in subpopulations after vaccination of children. Seven studies (8 publications) evaluated herd effect in household or family members [17,18,30-35]. The eighth study assessed herd effect in school contacts; however, point estimates were not reported in the paper but were recalculated from slopes [21]. Figure 3 shows the point estimates from these eight studies, with the lines derived from the point estimates from four studies of herd effect at a community level for comparison (twelve studies in all). The eight subpopulation studies share a general limitation, as the populations analysed for herd effect are restricted to household or family members or school contacts, who are still exposed to the risk of infection from the wider community without mass vaccination of a subpopulation [30]. In addition to the general limitation, the studies had particular methodological limitations (see Additional file 1 for details). Two studies failed to show any herd effect, which was explained by community exposure to infection of unvaccinated family members [30] or by low attack rates in household contacts, of whom 90% were adults with apparent partial immunity [33].

 Therefore, the studies evaluating herd effect at a community level were considered to provide better point estimates than the studies on herd effects in subpopulations, both because of methodological limitations in the subpopulation studies and the questionable ability to generalise their results to herd effects in the entire population. The minimum and maximum values from the study by Vynnycky et al. (2008) [29] for influenza A provided the most and least conservative results, with the point estimates from the other two community studies falling within the same range (Figure 3). The point estimates from the subpopulation studies also fell within the same range, if the two studies that failed to show any herd effect were disregarded (Figure 3). The Vynnycky et al. [29] point estimates have two potential limitations: firstly, the results considered were calculated for influenza A, and secondly, we included data only for the age group 15–44 years as analysed for herd effect. However, influenza A is the most common type of influenza, and results from the other studies, which did not differentiate between influenza A and B, were located within the same range. Variations between point estimates for herd effect in different age groups were small in all studies analysed, as the difference between specific age groups and the overall population analysed for herd effect did not exceed 7%. The impact of herd effect on different age groups is highly dependent on the contact pattern between age groups, and so differences between age groups may be relevant if contact patterns differ from the estimates considered in these studies. However, in the absence of more detailed data, it seems appropriate for the model population to use the same estimates for herd effect in the overall unvaccinated population in the model, without attempting to separate age groups.

### Estimating RR in the unvaccinated remainder of the age group targeted by childhood vaccination, as a function of effective coverage in that age group

**Figure 4:**
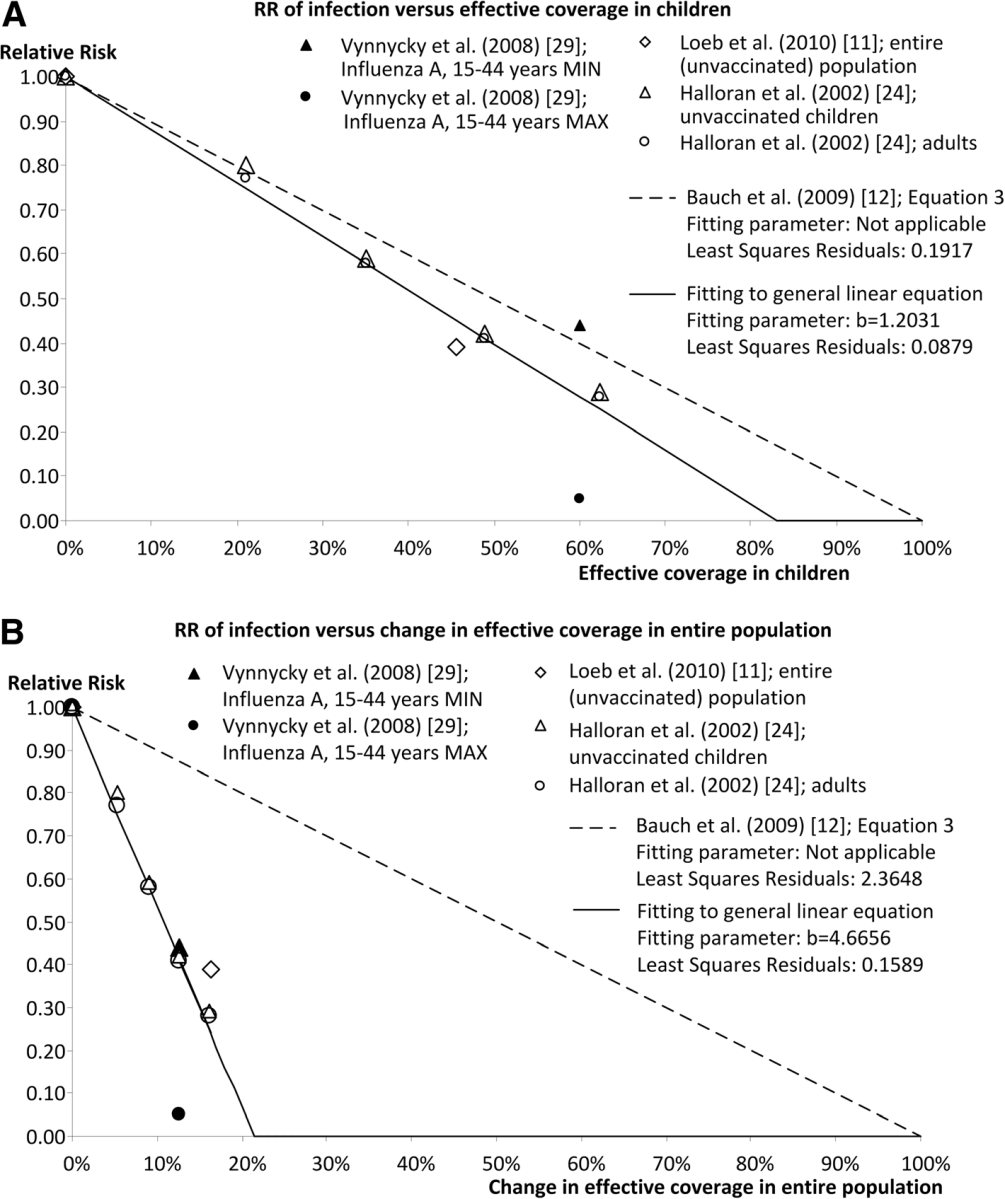
**Linear relationships between effective vaccine coverage and herd effect.** Point estimates identified from the literature review and linear relationships (derived from Equation 3 in Bauch et al. (2009) [12] or from fitting to general linear equation) between relative risk of infection in the unvaccinated population as a function of (**A**) effective coverage in children, and (**B**) change in effective vaccine coverage in entire population induced by varying levels of effective coverage in children.

The point estimates identified by the structured literature review as the best predictors of herd effect in the age group targeted by childhood vaccination are shown in Table 2 and Figure 4A. As the review did not identify evidence of substantial differences in point estimates between age groups, it is therefore assumed that the RR values are also applicable to unvaccinated children in the age group targeted by a childhood vaccination strategy. Effective coverage for this age group was calculated from vaccine efficacy and vaccination coverage in the target age group, as reported in the corresponding studies.

Figure 4A shows the results of fitting a general linear equation of the form y = a + bx to the point estimate data. A zero probability of infection (RR = 0) in the unvaccinated proportion of children occurs at an effective coverage of 83.1%. The slope of the fitted linear function is not very different to that of the linear function derived from Equation 3 of Bauch et al. (2009) [12].

These findings indicate that for this age group there are two possible approximations for estimating the indirect effect on the annual risk of infection that could be included in a static model. The approach derived from Equation 3 of Bauch et al. (2009) [12], which does not allow for fitting to the point estimate data, provides a more conservative estimate of herd effect:

$$RR_{\text{unvaccinated}\;\text{children}}=1-\text{effective}\;\text{coverage}\;\text{in}\;\text{children}$$

The approach derived from the linear equation fitted to the data from the literature review provides a slightly less conservative estimate of herd effect:

$$\begin{array}{l} RR_{\text{unvaccinated}\;\text{children}}=1-1.2031\\ \;*\;\text{effective}\;\text{coverage}\;\text{in}\;\text{children} \end{array}$$

With this equation, RR = 0 when effective coverage in children is higher than −1 / (− 1.2031), or 83.1%.

###  Estimating RR in other age groups, as a function of change in effective coverage in the entire population induced by varying levels of effective coverage in children

Table 2 and Figure 4 B show the point estimates identified in the literature review as the best predictors of herd effect in the other age groups (change in effective coverage of entire population, row B in Table 2). As the review was not able to identify evidence of substantial differences in point estimates between age groups, it is assumed that RR values are applicable to all age groups.

The effective coverage in the age groups not being targeted by the childhood vaccination strategy differs among the studies identified as best predictors, since most age groups were partially vaccinated in the base case or control group. As such, the RR values calculated during the literature review correspond to the change in effective coverage in the entire population induced by increasing effective coverage in children. For this reason, this change in effective coverage in the total population was recalculated, based on the age distribution applied in the corresponding studies (Table 2). As can be clearly seen in Figure 4B, a slight increase in effective vaccine coverage in the whole population, resulting from a programme of increasing vaccination coverage in children, results in a large decrease in RR of infection in the remainder of the community.

 Figure 4B shows the results of fitting a general linear equation to these data. In contrast to the results in the childhood population targeted for vaccination, there is a large difference between the fitted linear equation and the linear function derived from Equation 3 of Bauch et al. (2009) [12], with the latter being much more conservative (Figure 4B). With the fitted linear equation, RR = 0 when effective coverage in the total population is increased by 21.4%.

 For the age groups not targeted by the childhood vaccination strategy, there are two possible approximations for estimating the indirect effect on the annual risk of infection that could be included in a static model. The approach derived from Equation 3 of Bauch et al. (2009) [12], which does not allow for fitting to the point estimate data, provides a more conservative estimate of herd effect:

$$\begin{array}{l} RR_{\text{other}\;\text{age}\;\text{groups}}=1-\left(\text{effective}\;\text{coverage}\;\text{in}\;\text{children}\right)\\ \;*\;P_{\text{children}} \end{array}$$

where P_children_ is the proportion of children (i.e. the age groups targeted by a childhood vaccination strategy) in the total population.

 The approach derived from the linear equation fitted to the data from the literature review provides a more optimistic estimate of herd effect:

$$\begin{array}{l} RR_{\text{other}\;\text{age}\;\text{groups}}=1-4.6656\\ \;*\;\left(\text{effective}\;\text{coverage}\;\text{in}\;\text{children}\right)\\ \;*\;P_{\text{children}} \end{array}$$

With this equation, RR = 0 when the change in effective coverage in the entire population induced by effective coverage in children (effective coverage in children * P_children_) is higher than −1 / (−4.6656), or 21.4%, or – equivalently – if effective coverage in children is higher than 21.4% / P_children_.

## Discussion

Studies have shown that the potential benefit of vaccinating children against influenza extends to other members of their families, which supports the recommendation to make wider use of influenza vaccine in healthy children of any age in order to reduce the burden of infection on the community. The vaccination of otherwise healthy day-care and school-aged children may significantly reduce indirect influenza-related costs, thus supporting earlier economic modelling analyses of immunization programs [36]. The methods described in the present study allow an approximate assessment of this herd effect in traditional static models used in economic evaluation of annual vaccination of children against seasonal influenza. The estimation of herd effect is expressed as a function of effective coverage in children, a notion which combines both vaccine efficacy and coverage. As such, these approximations inherently incorporate the flexibility of estimating changes in magnitude of herd effect associated with varying levels of vaccination coverage in children, as well as for the impact of varying vaccine efficacy, which is – amongst others – dependent on the degree of strain matching and type of vaccine. A plausible range for the magnitude of this indirect effect can be estimated from the two methods of approximation identified in this research: (1) a general approach, irrespective of disease area, provides more conservative estimates, and (2) a data-driven approach, fitted to published data specific for influenza vaccination in children, provides less conservative estimates.

The structured literature review provided evidence to support the hypothesis of a linear relationship between effective coverage and RR within an effective coverage range (vaccine efficacy combined with coverage) of 20% to 80% of the subgroup targeted for vaccination. Point estimates identified from the literature review allowed the fitting of a linear equation of the form y = a + bx for each of two broad age groups, the age group targeted by a childhood vaccination strategy (i.e. children) and the group not targeted by the vaccination strategy (i.e. adults and/or elderly people). In children, the fitted equation was not very different from the slightly more conservative function derived from Equation 3 in Bauch et al. (2009) [12]. In the other age group, there was a large difference between the fitted linear equation and the linear function derived from Equation 3 of Bauch et al. (2009) [12], with the latter being much more conservative. Thus, using the linear approximations derived from Equation 3 in Bauch et al. (2009) [12] for both age groups would provide a conservative estimate of herd effect, while using the linear functions fitted to data from this structured literature review would provide a less conservative estimate of herd effect. Both approximations require only simple adjustments to the annual baseline risk of influenza for the two age groups, and can therefore be easily incorporated into static models to provide an approximate estimate of the likely range of possible herd effects.

### Limitations

A non-dynamic approximation such as those presented here cannot replace a fully dynamic modelling approach, and should only be intended for a preliminary assessment of herd effect [12]. However, the linear approximations derived from Equation 3 in Bauch et al. (2009) [12] are considered by the respective authors to be more conservative than a full dynamic assessment. Our second linear approximation was fitted to point estimates that included estimates derived from dynamic models, and can therefore be considered as more closely mimicking a full dynamic assessment of herd effect (which is the ultimate objective of a non-dynamic approximation). This second approximation offers a method for making a less conservative estimate of herd effect, and should thus help to allow a fuller exploration of the potential impact of herd effect within a static model.

Our second linear approximation is only intended for exploratory purposes, since it implicitly assumes a constant basic reproduction number (R_0_) for seasonal influenza. The potential bias induced by this assumption is likely to be marginal for seasonal influenza, since R_0_ estimates for these epidemics are low and fairly constant [37]. However, as a consequence of this assumption, our second linear approximation cannot as such be applied to provide a preliminary assessment of potential herd effect in pandemic situations.

Although the literature review conducted was not systematic, it was structured in a transparent and reproducible manner, with search terms, eligibility criteria and data extraction defined in advance. An independent reviewer checked all included studies and data extracted, in an effort to minimise selection bias. However, the initial screening process included studies that could not be ruled out with certainty, and reasons for exclusion were documented for all studies rejected after full text review. In addition, the inclusion of studies from sources other than the database search (in this review, mainly from reference lists) also bears a risk of selection bias. Most of the studies identified as useful for the main aim of the project were derived from the database search, and the two which came from other sources [21,23] reported outcomes that did not differ from the other studies.

The literature review did not reveal a mathematical function for the relationship between the relative risk in unvaccinated and very low (<20%) or very high (>81.1%) effective coverage levels in a subpopulation. However, findings have indicated that herd effect is relevant even with very low levels of coverage and can be even greater than direct effect [13]. This finding is supported by other authors, who reported that the extent to which the elderly benefit from indirect effects depends (among other factors) on disease transmissibility [38]. Below a certain transition point, the elderly were protected more by the indirect effects of the morbidity-based strategy than by direct effects of the mortality-based strategy [38]. Accordingly, in epidemics a relevant indirect effect can also be assumed for very low levels of effective coverage, and can even be higher than the direct effects [13]. However, this is highly dependent on the transmissibility, which is linearly related to R_0_[38].

For very high levels of effective coverage, i.e. very high coverage and vaccine efficacy, a linear function might overestimate the impact of herd effect and a flattening of the curve, i.e. a more exponential function with exponent <1 in age groups others than those considered for mass vaccination might be expected. However, this is a more intuitive conclusion, rather than based on evidence from literature search.

Depending on the study, the RR of infection was calculated from either the probability of infection (modelling studies) or the probability of symptomatic influenza (observational studies). Thus, we implicitly assumed that both probabilities are linearly related, so that the RR is identical irrespective of which outcome is considered. It is however important to note that the RR obtained with our approximations refer to the baseline risk of true influenza infections (whether or not symptomatic), and do not reflect the reduction in risk of influenza-like-illness (ILI). Seasonal influenza vaccination is not efficacious against ILI other than true influenza, and hence will only partially reduce transmission of all ILI. As such, the effective coverage estimates to be applied in our linear approximations should be based on vaccine efficacy against true influenza, and not vaccine effectiveness against ILI. And consequently, our approximations can only be applied in cohort models operating on the basis of true influenza and its health and economic consequences.

Our second linear equation fitted to the point estimates in this literature review assumes that individuals in a population mix randomly within and between all age groups, and do not take account of the variety of mixing and contact patterns apparent in real life. The wide range between the minimum and maximum point estimates derived from Vynnycky et al. (2008) [29] clearly demonstrates the impact of different mixing contact patterns on the size of the indirect effects of vaccination. Empirical data such as the POLYMOD contact survey [39] indicate that mixing between age groups is often highly assortative, i.e. people have contacts primarily with people of the same age group as themselves. Thus, this assumption of random mixing, inherent to our second linear approximation, is likely to overestimate the importance of herd effect on age groups other than those targeted by vaccination in communities with a relatively low inter-age mixing (e.g. communities with low frequency of multi-generational households).

A further limitation is that the approximation of herd effect in age groups not targeted for vaccination does not account for any effective vaccine coverage already present in those age groups. If effective coverage is already substantial in these age groups, a modest increase in effective coverage in the total population induced by vaccinating children might result in a situation where the elimination threshold is exceeded and RR falls to zero. As such, the magnitude of the herd effect reported by the studies identified in this review is dependent on the pre-existing vaccine coverage in the age groups not targeted for vaccination. This could explain why the point estimate derived from the study by Loeb et al. (2010) [11] was less favourable than the other studies shown in Figure 4B. In the study by Loeb et al. (2010) [11] vaccination coverage in the remainder of the population was quite low (<13%), whereas in Halloran et al. (2002) [24] 22.9% of adults aged 19–64 years and 68.1% of the elderly were vaccinated. Consequently, the less conservative linear function, derived by fitting to these point estimates, is likely to overestimate herd effect in groups that have little or no vaccine coverage.

For the purpose of this study, point estimates of effective coverage were derived or calculated from vaccine efficacy data reported in the various publications. There is a risk of bias when using data from observational studies since the vaccinated population might also potentially benefit from a reduction in the baseline risk of influenza (indirect effect), where observed vaccine efficacy is in fact the sum of both direct and indirect effects of vaccination. However, the linear fitting in our study was performed against data extracted from three publications in which this risk of bias is not present or negligible: the two modelling studies compared the post-vaccination population against a pre-vaccination population [24,29], and the vaccine efficacy reported in the one observational study statistically corrected for this bias [11]. However, this aspect needs to be considered thoroughly, in case future studies are included in the fitting process in further research.

As a result of these limitations, we would recommend using the more conservative approach (the linear function derived from Equation 3 of Bauch et al. (2009) [12]) as the base case for cost-effectiveness analyses using a static model. We would recommend using the less conservative approach, using the linear functions fitted to the point estimates in this literature review, in sensitivity analyses. The less conservative approach may overestimate the effects of herd effect induced by childhood vaccination, particularly for age groups with a low likelihood of mixing with children and/or with little or no pre-existing vaccination coverage. However, it allows a fuller exploration of the potential impact of herd effect than the conservative approach alone. Both approximations require only simple adjustments to be made to the annual baseline risk of influenza for the two age groups, and can therefore be incorporated into static models. They can be used together to explore the likely range of herd effects in static models, without requiring dynamic modelling processes.

## Conclusions

This method of approximating herd effect does not rely on dynamic modelling and can be used in static models. It requires simple adjustments to the annual baseline risk of influenza, first for the age group targeted by the childhood vaccination strategy (i.e. children), and second for other age groups not targeted by vaccination (e.g. adults and/or elderly people). We present two approximations that provide a linear relationship between effective coverage and reduction in the risk of infection. The first is a conservative approximation, recommended as base-case for cost-effectiveness evaluations. The second, fitted to data extracted from a structured literature review, provides a less conservative estimate of herd effect and is recommended for use in sensitivity analyses.

## Abbreviations

RR: Relative risk; SSR: Sum of squared residuals; WHO: World Health Organization; ILI: Influenza-like-illness.

## Competing interests

GM is an employee of GlaxoSmithKline group of companies and holds stock or stock options in GlaxoSmithKline group of companies.

IVV’s and LAVB’s institution received consulting fees from GlaxoSmithKline Biologicals SA for conducting the present study and writing the manuscript outline, and has also received consultancy fees from GlaxoSmithKline Biologicals SA for other projects and for writing manuscript outlines related to these other projects.

BNP received consulting fees for conducting the literature review and fees for writing the manuscript outline from GlaxoSmithKline Biologicals SA in relation to the present study.

## Authors’ contributions

IVV, LAVB and GM designed the study, BPN conducted the literature review, IVV was an independent reviewer of the literature review. IVV and LAVB carried out the mathematical analyses and fitting of the linear equations. All authors reviewed and commented on manuscript drafts, and read and approved the final manuscript.

## Pre-publication history

The pre-publication history for this paper can be accessed here:

http://www.biomedcentral.com/1471-2334/13/25/prepub

## Supplementary Material

Additional file 1 Additional details of published studies. Click here for file
