# Supplementary material for: An approximation of herd effect due to vaccinating children against seasonal influenza – a potential solution to the incorporation of indirect effects into static models
Source: BMC Infect Dis. 2013 Jan 22;13:25. doi: 10.1186/1471-2334-13-25 (PMC3570298; doi:10.1186/1471-2334-13-25)
Supplement: Additional file 1 — Additional details of published studies. [file 1471-2334-13-25-S1.doc]

# Additional File 1

# Results

## Details of studies reporting data useful for the estimation of a mathematical function between varying degrees of effective coverage in subgroup populations and the reduction of risk of influenza infections in a larger unvaccinated population

Of the six studies included, two provided a graphical illustration [1,2] and three reported data that allowed the estimation of a graphical illustration [3-5]. One study provided other relevant data [6].

Van den Dool et al used a dynamic population model to simulate the impact of mass vaccination of healthcare workers in Dutch nursing homes on the incidence of influenza in nursing home patients [2]. The authors found a strong linear relationship between patients’ attack rates and varying levels of vaccination coverage in healthcare workers from 0% to 100% (recalculated from data reported to a range of effective coverage between 30% and 81.1%). The authors reported a relative risk of infection in patients of 0.41 when all healthcare workers were vaccinated, compared with no vaccination in healthcare workers. The authors also conducted numerous scenario analyses, all resulting in linear relationships between vaccination coverage and patients’ attack rates, including one scenario analysis assuming ‘no vaccination’ (zero effectiveness of vaccine) in patients, and one analysis considering a nursing home setting comparable to an open department, i.e. corresponding to the entire community.

A prospective cluster-controlled trial found significant slopes for the percentage of children vaccinated with live attenuated influenza vaccine (LAIV) and staff illness (slope –1.47), and the percentage of children vaccinated with LAIV and illness rate of unvaccinated students in the same school (slope –1.15) [1]. When recalculating effective coverage from the data reported, the linear relationship corresponded to a range between 7.3% and 29.9% effective coverage (vaccine effectiveness was reported to be most likely underestimated, accordingly the same can be assumed for effective coverage).

Three dynamic population models [3-5] estimated indirect effects at varying levels of vaccination coverage, which allowed recalculation to a graphical relationship between effective coverage and RR of infection. Two dynamic population models [3,4] resulted in linear relationships over the range of vaccine coverage reported in the studies, and one dynamic population model [5] resulted in an exponential function (with exponent <1) for a range of effective coverage between 3.5% and 56%.

One additional study [6] provided data on the proportion of indirect effects from the overall effect of vaccination with low and medium levels of coverage. The authors developed two types of models, a static and a dynamic model (not validated against real life data) simulating the progress of an influenza epidemic in Spain, with the difference between the two models being attributed to herd effect. The data allowed a recalculation of the effective coverage for the three scenarios considered (less favourable, basal, more favourable), and the calculation of the percentage of cases avoided that could be attributed to indirect protection. With low levels of effective coverage a high percentage of the total vaccination effect was estimated to be due to herd effect. With an effective coverage of 2.8%, 84.6% of all cases avoided were estimated to be due to herd effect; however, the proportion decreased with increasing levels of coverage (effective coverage of 13.4% and 50.4% resulting in percentages of 76.8% and 35.4% of all cases avoided being due to herd effect, respectively).

## Details of studies reporting point estimates for the reduction of influenza infection in the unvaccinated population after vaccination of children

### Studies evaluating a herd effect on a community level

Of the studies evaluating herd effect at a community level, five were considered unsuitable for estimation of point estimates. Three studies [7-9] were all part of the Central Texas Field Trial and used the outcome parameter ‘medically attended acute respiratory illness’ (MAARI). MAARI has considerable limitations, since not all people with influenza are seeking medical attendance, and people who are vaccinated may experience less serious illnesses, tending not to consult a physician. On the other hand not every MAARI is due to influenza. One of these studies [9] reported similar vaccination rates (except for the age group eligible for vaccination) between populations analyzed for herd effect in intervention and comparison sites, but Figure 1 of the paper indicated differences in vaccination coverage, suggesting that differences in risk reduction are most likely to be due to combined direct and indirect effects. In addition, there were uncertainties regarding vaccine effectiveness, which was reported inconsistently for laboratory-confirmed influenza in the intervention and comparison sites with very small sample sizes (intervention site RR [vaccines combined] = 0.25, comparison site RR [vaccines combined] = 0.85). The direct effectiveness for number of MAARI events per week was calculated to RR = 0.8734 with the live attenuated vaccine. The other two studies in this group [7,8] failed to document any direct protective effectiveness in vaccinated children compared to non-vaccinated children, which also limits the generalizability of any findings on indirect effects of the vaccine. Another of the five excluded studies only allowed the recalculation of RR to a reference (RR = 1.00) that corresponded to 3.5% effective coverage rather than zero coverage [5], and the fifth study was validated for pandemics rather than epidemics [3].

Three studies were considered suitable for the identification of point estimates on the reduction of influenza incidence in the unvaccinated population after vaccination of children [4,10,11].

The study by Loeb et al [10] is the only cluster randomized trial conducted on a community level with laboratory-confirmed influenza illness. Investigators and surveillance nurses were blind to allocation status. Mass vaccination against influenza in children aged 36 months to 15 years was performed in 22 Hutterite colonies, whereas children in 24 Hutterite colonies received Hepatitis A vaccine, representing the control communities. Indirect effectiveness in other members of Hutterite colonies was adjusted for out-of-study vaccination, and totalled 61% (recalculated to a relative risk of 0.39). Data reported allowed recalculation to an effective coverage of 45.65%. The study has a number of limitations: demographic differences between Hutterite colonies and general populations; questionable transferability of the contact patterns in the isolated Hutterite colonies to the general population; and direct effectiveness as measured in the study failed to reach statistical significance.

Vynnycky et al. [11] reported a dynamic population model simulating the impact of vaccinating children on the reduction of the incidence of influenza in individuals of different age groups in the UK. We considered only the results presented for mass vaccination of the age group 6 months to 16 years, and for herd effect in the age group 15-44 years, since other populations analysed for herd effects were part of or included the subgroup of vaccinated children. Results were presented as ranges (minimum and maximum), resulting from simulations of different contact patterns. Results were estimated for R_0_ = 1.8, which is a more conservative approach than results for R_0_ = 1.3. Limitations of the study included the restricted age group (age 15-44 years) considered for the analysis of herd effect, and the fact that only results calculated for influenza A were considered, as the reported results for influenza B were not disaggregated in age groups and included the subgroup of vaccinated children.

Halloran et al. [4] also reported a dynamic population model, simulating the effect of vaccinating children and adolescents aged 1-18 years on the reduction of the incidence of influenza in individuals of different age groups in the US. The population analysed for herd effects included unvaccinated children and adults (partly vaccinated, but vaccine coverage was kept constant). The study was not validated against real-life data.

### Studies evaluating a herd effect in a subpopulation

Eight studies provided information on herd effects in subpopulations after vaccination of children. Seven studies (8 publications) evaluated herd effect in household or family members [12-19]. The eighth study assessed herd effect on school contacts; however, point estimates were not reported in the paper but were recalculated from slopes [1]. These studies share a general limitation, as the populations analysed for herd effect are restricted to household or family members or school contacts, who are still exposed to the risk of infection from the wider community without mass vaccination of a subpopulation [12]. In addition to the general limitation, the studies had particular methodological limitations. Two studies failed to show any herd effect, which was explained by community exposure to infection of unvaccinated family members [12] or by low attack rates in household contacts, of whom 90% were adults with apparent partial immunity [15]. Three studies (four publications) probably underestimated vaccine effectiveness and consequently effective coverage [1,14,17,18]. In two studies [13,16] vaccination status deviated between vaccine and control groups, so that effects measured could not be attributed to indirect effects only, and one study did not report vaccination status in the populations [19].

# References

1. Rudenko LG, Slepushkin AN, Monto AS, Kendal AP, Grigorieva EP, Burtseva EP *et al*.: **Efficacy of live attenuated and inactivated influenza vaccines in schoolchildren and their unvaccinated contacts in Novgorod, Russia.** *J Infect Dis* 1993, **168:** 881-887.

2. van den Dool C, Bonten MJ, Hak E, Heijne JC, Wallinga J: **The effects of influenza vaccination of health care workers in nursing homes: insights from a mathematical model.** *PLoS Med* 2008, **5:** e200.

3. Elveback LR, Fox JP, Ackerman E, Langworthy A, Boyd M, Gatewood L: **An influenza simulation model for immunization studies.** *Am J Epidemiol* 1976, **103:** 152-165.

4. Halloran ME, Longini IM, Cowart DM, Nizam A: **Community interventions and the epidemic prevention potential.** *Vaccine* 2002, **20:** 3254-3262.

5. Weycker D, Edelsberg J, Halloran ME, Longini IM, Jr., Nizam A, Ciuryla V *et al*.: **Population-wide benefits of routine vaccination of children against influenza.** *Vaccine* 2005, **23:** 1284-1293.

6. Pradas-Velasco R, Antonanzas-Villar F, Martinez-Zarate MP: **Dynamic modelling of infectious diseases: an application to the economic evaluation of influenza vaccination.** *Pharmacoeconomics* 2008, **26:** 45-56.

7. Piedra PA, Gaglani MJ, Kozinetz CA, Herschler G, Riggs M, Griffith M *et al*.: **Herd immunity in adults against influenza-related illnesses with use of the trivalent-live attenuated influenza vaccine (CAIV-T) in children.** *Vaccine* 2005, **23:** 1540-1548.

8. Piedra PA, Gaglani MJ, Kozinetz CA, Herschler GB, Fewlass C, Harvey D *et al*.: **Trivalent live attenuated intranasal influenza vaccine administered during the 2003-2004 influenza type A (H3N2) outbreak provided immediate, direct, and indirect protection in children.** *Pediatrics* 2007, **120:** e553-e564.

9. Glezen WP, Gaglani MJ, Kozinetz CA, Piedra PA: **Direct and indirect effectiveness of influenza vaccination delivered to children at school preceding an epidemic caused by 3 new influenza virus variants.** *J Infect Dis* 2010, **202:** 1626-1633.

10. Loeb M, Russell ML, Moss L, Fonseca K, Fox J, Earn DJ *et al*.: **Effect of influenza vaccination of children on infection rates in Hutterite communities: a randomized trial.** *JAMA* 2010, **303:** 943-950.

11. Vynnycky E, Pitman R, Siddiqui R, Gay N, Edmunds WJ: **Estimating the impact of childhood influenza vaccination programmes in England and Wales.** *Vaccine* 2008, **26:** 5321-5330.

12. Clover RD, Crawford S, Glezen WP, Taber LH, Matson CC, Couch RB: **Comparison of heterotypic protection against influenza A/Taiwan/86 (H1N1) by attenuated and inactivated vaccines to A/Chile/83-like viruses.** *J Infect Dis* 1991, **163:** 300-304.

13. Esposito S, Marchisio P, Cavagna R, Gironi S, Bosis S, Lambertini L *et al*.: **Effectiveness of influenza vaccination of children with recurrent respiratory tract infections in reducing respiratory-related morbidity within the households.** *Vaccine* 2003, **21:** 3162-3168.

14. Ghendon YZ, Kaira AN, Elshina GA: **The effect of mass influenza immunization in children on the morbidity of the unvaccinated elderly.** *Epidemiol Infect* 2006, **134:** 71-78.

15. Gruber WC, Taber LH, Glezen WP, Clover RD, Abell TD, Demmler RW *et al*.: **Live attenuated and inactivated influenza vaccine in school-age children.** *Am J Dis Child* 1990, **144:** 595-600.

16. Hurwitz ES, Haber M, Chang A, Shope T, Teo S, Ginsberg M *et al*.: **Effectiveness of influenza vaccination of day care children in reducing influenza-related morbidity among household contacts.** *JAMA* 2000, **284:** 1677-1682.

17. Monto AS, Davenport FM, Napier JA, Francis T, Jr.: **Effect of vaccination of a school-age population upon the course of an A2-Hong Kong influenza epidemic.** *Bull World Health Organ* 1969, **41:** 537-542.

18. Monto AS, Davenport FM, Napier JA, Francis T, Jr.: **Modification of an outbreak of influenza in Tecumseh, Michigan by vaccination of schoolchildren.** *J Infect Dis* 1970, **122:** 16-25.

19. Principi N, Esposito S, Marchisio P, Gasparini R, Crovari P: **Socioeconomic impact of influenza on healthy children and their families.** *Pediatr Infect Dis J* 2003, **22:** S207-S210.
